# Accuracy vs. Energy: An Assessment of Bee Object Inference in Videos from On-Hive Video Loggers with YOLOv3, YOLOv4-Tiny, and YOLOv7-Tiny

**DOI:** 10.3390/s23156791

**Published:** 2023-07-29

**Authors:** Vladimir A. Kulyukin, Aleksey V. Kulyukin

**Affiliations:** Department of Computer Science, Utah State University, Logan, Utah, USA

**Keywords:** precision apiculture, precision pollination, computer vision, artificial intelligence, deep learning, energy efficacy, power efficiency, YOLO, *Apis mellifera*, honey bee, hive monitoring

## Abstract

A continuing trend in precision apiculture is to use computer vision methods to quantify characteristics of bee traffic in managed colonies at the hive’s entrance. Since traffic at the hive’s entrance is a contributing factor to the hive’s productivity and health, we assessed the potential of three open-source convolutional network models, YOLOv3, YOLOv4-tiny, and YOLOv7-tiny, to quantify omnidirectional traffic in videos from on-hive video loggers on regular, unmodified one- and two-super Langstroth hives and compared their accuracies, energy efficacies, and operational energy footprints. We trained and tested the models with a 70/30 split on a dataset of 23,173 flying bees manually labeled in 5819 images from 10 randomly selected videos and manually evaluated the trained models on 3600 images from 120 randomly selected videos from different apiaries, years, and queen races. We designed a new energy efficacy metric as a ratio of performance units per energy unit required to make a model operational in a continuous hive monitoring data pipeline. In terms of accuracy, YOLOv3 was first, YOLOv7-tiny—second, and YOLOv4-tiny—third. All models underestimated the true amount of traffic due to false negatives. YOLOv3 was the only model with no false positives, but had the lowest energy efficacy and highest operational energy footprint in a deployed hive monitoring data pipeline. YOLOv7-tiny had the highest energy efficacy and the lowest operational energy footprint in the same pipeline. Consequently, YOLOv7-tiny is a model worth considering for training on larger bee datasets if a primary objective is the discovery of non-invasive computer vision models of traffic quantification with higher energy efficacies and lower operational energy footprints.

## 1. Introduction

Honey bee (*Apis mellifera*) traffic in the hive’s vicinity is a contributing factor to the hive’s productivity and health [1], which is why attempts to quantify it with human observations and mechanical, analog, and digital sensors have continued for almost a century [2]. One such sensor is a camera, a non-invasive sensor that, when placed on or outside of the hive, does not disrupt the colony’s natural cycles or require significant structural modifications of the hive. In 1935, Patterson [3] designed a photo-based bee counter with a conventional camera with a wide-angle lens and 35 mm film. Single bee passes were manually counted in individual photos as line crossings. Patterson’s system required approximately 20 m of film per minute. For the next 60 years, computer vision methods mostly disappeared from insect motion research until the appearance of affordable digital cameras in the late 1990s, which allowed researchers to capture not only still images, but also videos to investigate insect motion (e.g., [4,5]).

The availability of affordable digital cameras prompted multiple precision apiculture researchers to start working on computer vision methods to investigate various types of traffic at the hive’s entrance or in the hive’s vicinity. Kimura et al. [6] proposed a method to count bees and measure their motion on internal frames of an observation hive. Chen et al. [7] developed a method to use an infrared camera to recognize the identity and orientation of individual bees marked with circular paper tags with special characters at the hive’s entrance. Dussaubat et al. [8] designed an optic bee counter system that used a digital camera looking down on the entrance of a 4-frame nuc hive. Chiron et al. [9] proposed a system to detect and track honey bees at the hive’s entrance with 3D stereo vision methods. Ghadiri [10] proposed a method to count individual bees in videos from on-hive loggers by using background segmentation in image sequences. Babic et al. [11] designed a pollen-bearing forager counting system that ran *in situ* on a Raspberry Pi 2 computer coupled to a Raspberry Pi High-Definition (HD) camera. Tu et al. [12] proposed a system for counting bees at the hive’s entrance and measuring incoming and outgoing traffic. Ngo et al. [13] designed and deployed a pollen-bearing forager counting system for 4-frame nuc hives. Tashakkori et al. [14] proposed Beemon, an integrated multi-sensor data acquisition and hive monitoring platform where videos from on-hive loggers are sent to a remote server for analysis. Kulyukin et al. [15] designed BeePIV, an algorithm that uses particle image velocimetry methods [16] to estimate the amount of incoming, outgoing, and lateral traffic in videos from on-hive loggers.

A recent trend in computer vision (CV) is the employment of methods of artificial intelligence (AI), a branch of computer science (CS) whose objective is to design algorithms that model natural intelligence. Deep learning (DL) is a branch of AI that focuses on the design and application of convolutional neural networks (ConvNets) to problems that include, but are not limited to, image generation and classification, speech and audio processing, and music synthesis and analysis [17]. ConvNets have also been used to detect Varroa mites on bees [18] and to classify different bumble bee species with smartphones [19].

While there is a growing appreciation that DL and other machine learning (ML) models are data- and energy-hungry [20], we are not aware of longitudinal precision apiculture studies that discuss in depth, let alone quantify, tradeoffs between the performance accuracies and energy footprints of such models. Yet, such investigations are urgently needed because of rising energy costs. For example, since fall 2022, the city of Logan, Utah, USA, home of Utah State University and one of the research sites for this investigation, has levied an electrical surcharge on monthly utility bills “to recover costs Logan City incurred as it purchased electricity on the open market where prices were higher than expected for reasons that were not foreseeable.” [21]. Such investigations are also fundamental because of the rising ecological and environmental costs of cloud computing required to support the continuous operation of many DL and ML models. Some of the prominent environmental and ecological costs are growing water consumption rates for cooling server farms [22], rising ocean water temperatures due to submerged data centers [23], rising ambient temperatures due to the heat generated by server farms [24], and increasing levels of electromagnetic radiation potentially harmful to honey bees and other animals [25,26].

To this end, we assessed the potential of three open-source ConvNet models, YOLOv-3 [27,28], YOLOv4-tiny [29], and YOLOv7-tiny [30], to quantify omnidirectional bee traffic in videos not only in terms of their accuracy, i.e., their estimated capacity to infer flying *Apis mellifera* objects in video frames, but also in terms of their *energy efficacy* and *operational energy footprint*. By the former, we mean a measurement of the number of performance units per every unit of energy required to make a model operational in a data pipeline; by the latter—the energy footprint of a functioning data pipeline over a period of time after a model is integrated into it. We made our assessments with BeePiP (pronounced as *bee pipe*), a multi-sensor continuous hive monitoring data pipeline (CHMDP) that we have been investigating since 2017 (e.g., [31,32]).

The contributions of our investigation can be summarized as follows.

1. **Image Dataset**: We curated an image dataset of 23,173 flying bees manually labeled in 5819 video frames from 10 randomly selected low-end, low-energy on-hive camera videos (744 frames per video) from colonies housed in regular, unmodified one- and two-super Langstroth hives with different queen races at different apiaries at different years.
2. **YOLO Models:** We trained and tested YOLOv3, YOLOv4-tiny, and YOLOv7-tiny with a 70/30 split on the curated dataset to infer flying bee objects in low-end, low-energy on-hive camera videos.
3. **Accuracy Analysis**: We manually evaluated the trained models on 3600 images from 120 randomly selected low-end, low-energy on-hive camera videos from apiaries different from the apiaries of the image dataset with different queen races at different years and performed a standard accuracy analysis of the models based on our evaluations in terms of true and false positives and true and false negatives. Our evaluation of bee object inference accuracy was based on seven evaluation categories that we specifically designed for this investigation.
4. **Energy Efficacy Analysis**: We designed a new energy efficacy metric to estimate the number of performance units per every unit of energy to make a model operational in a data pipeline and computed the energy efficacy coefficients for each performance accuracy metric and each evaluated model.
5. **Operational Footprint Analysis**: We performed an analysis of the energy footprint of BeePiP deployed at an apiary in Logan, Utah, USA with each trained model integrated into it over different time periods.
6. **Open Science**: We made our bee image dataset and the three best-trained YOLO models publicly available for replicability and iterative improvement in the supplementary materials.

## 2. Materials and Methods

### 2.1. Continuous Hive Monitoring Data Pipeline

**Figure 1:**
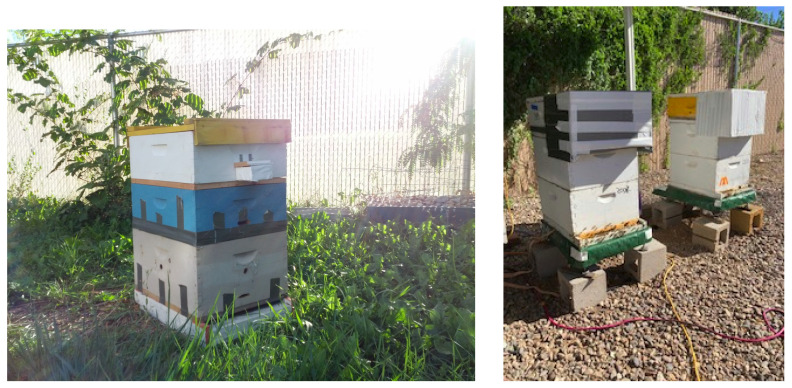
**Left**: An on-hive BeePi logger on top of a 2-super Langstroth hive in Logan, Utah; bottom to top: (1) a landing pad; (2) a light gray super; (3) a blue super; (4) a white super with the BeePi logger hardware (see the left image in Figure 2); (5) a waterproof plastic box with a Pi camera inside (see Figure 3) looking down on the landing pad; the box is attached to the front of the third super with two screwed metallic brackets; (6) a wooden migratory hive lid on top of the third white super. **Right**: two BeePi loggers on top of two super Langstroth hives in Tucson, Arizona; the top boxes on hives contain the logger hardware; water- and dustproof boxes on top of the second supers protect the cameras against rain and dust storms frequent in that area of Arizona.

**Figure 2:**
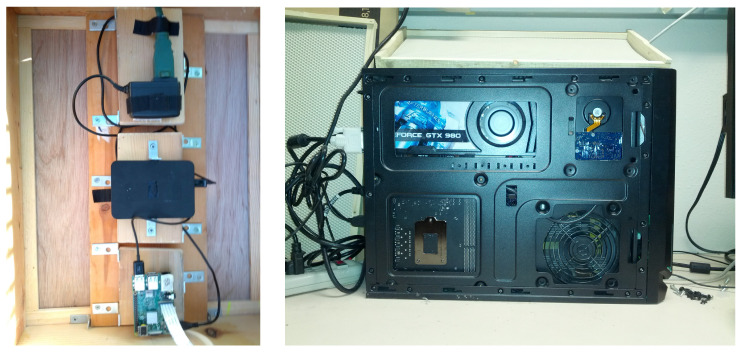
**Left**: BeePi logger hardware; bottom to top: a Raspberry Pi computer coupled to an 8-megapixel Pi camera (see left image in Figure 3); a five terabyte USB disk for archiving data for redundancy in case of GPU computer failures or power supply disruptions; a Pi power charger plugged into a waterproof power cord; videos are wirelessly transferred to a GPU computer over an ad hoc 802.11 local network, where they are processed and archived for redundancy in case of logger storage failures. **Right**: GEFORCE GTX-980 GPU computer (Arc: x86_64; CPU Family: 6; Model: 60; Model Name: Intel(R) Core(TM) i7-4790K; CPU at 4.00 GHz; BogoMips: 7999.890) with Ubuntu 18.04; all YOLO models were trained on this computer and evaluated for their energy efficacy and operational energy footprint.

**Figure 3:**
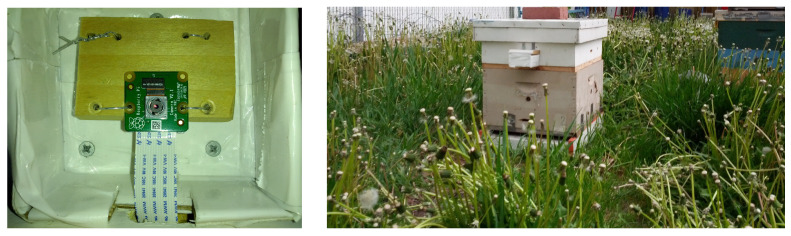
**Left**: A low-end, low-energy 8-megapixel Raspberry Pi camera v2.1 inside a waterproof camera protection box attached to the front of the super with the on-hive BeePi logger shown in the right picture. **Right**: An on-hive BeePi logger on top of a one super hive in Logan, Utah in May 2023; bottom to top: (1) a bottom board with a landing pad; (2) a light gray super; (3) a white super with the logger hardware shown in the left image of Figure 2, and a white waterproof camera protection box with the Pi camera in the left image; (4) a white telescoping hive lid.

All videos for our study were captured within the framework of BeePiP. When installed at an apiary, a BeePiP consists of BeePi on-hive loggers, one logger per hive (see Figure 1, Figure 2 and Figure 3), and a local GPU computer where the wirelessly transferred data from the loggers are processed. The GPU computer used in this investigation is shown in the right image of Figure 2. While in this article, we focus on bee object inference in videos, BeePi loggers collect other data relevant to hive monitoring such as hive weight, internal hive temperature, weather, and ambient electromagnetic radiation. Since 2017 we have deployed BeePiP at five public and private apiaries in Utah, Arizona, and California (see Table 1). We varied the number of loggers from 2 to 10 and the GPU computer with no monitor or keyboard was placed in a nearby building, i.e., a barn, a garage, or a storage shed, sufficiently close to the on-hive loggers for wireless data transfer over an ad hoc 802.11 local network.

The loggers are powered around the clock through waterproof extension cords plugged into electrical outlets on walls of nearby buildings or on solar stations. Video logging is parameterized by the duration of each video (e.g, 10 s), the video capture frequency (e.g, every 5 min), and the daily video capture period (e.g, 7:30 to 20:30). These parameters are adjustable through configuration files to fit specific project requirements, e.g., local climates, power supply limitations, manual hive inspection schedules, etc. The loggers are completely non-invasive in that they require no structural modifications of the hives, e.g., special tube tunnels for bee entrance or exit, transparent plastic boards on top of landing pads to make foragers and drones crawl in and out of the hive, or any sensors or special markings on individual bees.

Videos wirelessly transferred by loggers to the GPU computer are processed with the OmniBeeM [31] and BeePIV [15] algorithms. Since the experiments reported in this investigation were done within the OmniBeeM framework, we will briefly describe it here. OmniBeeM stands for **Omni**directional **Bee** Motion. The algorithm consists of three logical components: object inference, motion detection, and object-motion alignment. The first two components operate in parallel or in sequence. When bee object inference must be informed by motion, motion detection is done prior to bee object inference with the latter applied only to detected motion regions, and, vice versa, motion detection can be confined to the regions with inferred bee objects. Object-motion alignment is a method of determining which motion regions and inferred bee objects constitute a *flying bee*. The output of OmniBeeM for each video is a non-negative integer representing the number of flying bee objects inferred in the video’s frames. OmniBeeM is agnostic to the specifics of object inference, motion detection, and object-motion alignment methods, and is designed as a software testbed for different combinations that are executed concurrently or sequentially.

### 2.2. Datasets

All videos for this investigation were captured by loggers equipped with low-end, low-energy 8-megapixel Pi v2.1 cameras connected to Raspberry Pi computers (Pi 3 Model B+ or Pi 4) running the Raspbian Operating System (OS), a flavor of open-source Debian OS. Each logger’s computer had a miniature heat sink on its CPU and was connected to exactly one Pi camera and to a digital clock (ChronoDot 2.1 Real-Time Clock, Macetech, Inc.) for video timestamping. At each apiary, the loggers captured 30-second (30-s) MPEG-4 (MP4) videos with a resolution of 1080 × 1980 pixels and a rate of 24 frames per second (fps) every 15 min daily from 7:30 to 20:45 in the local time zone. When the logger is installed on the first super, the camera’s height (CH), i.e., the distance from the landing pad to the camera’s lens is 30 cm, and the volume of the space, i.e., height by width by length, in front of the hive in recorded videos is approximately 30 cm × 50 cm × 70 cm. When installed on the second super, the camera’s height is 60 cm, and the volume is approximately 60 cm × 65 cm × 85 cm. We refer to videos recorded with loggers on the first super as CH=1 and to videos from loggers on the second super as CH=2.

**Figure 4:**
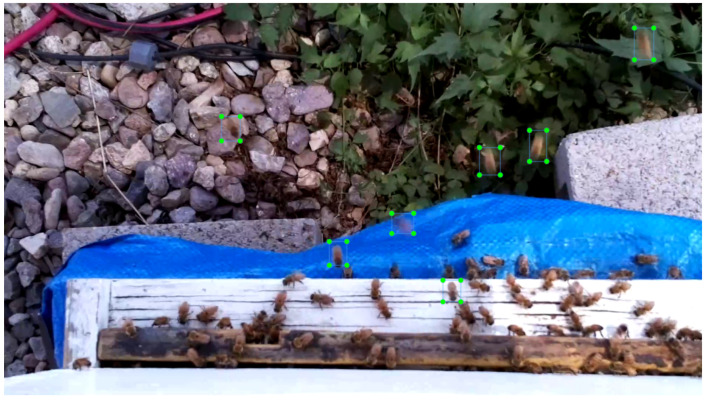
Manually labeled flying bees in a frame from a video captured by a BeePi logger on top of a 2-super hive at an apiary in Tucson, Arizona in September 2022. Each bee object is marked with a rectangle with corners accentuated by small filled light-green circles. When we were not sure if an object is a bee shadow or a bee, we left it unlabeled. Nor did we label any partial bee objects (1/2 bee, 1/3 bee, etc.) or any bee object that we could not recognize, e.g., due to the fogging of a camera lens or video flicker caused by wind. Since our objective was to develop YOLO models to quantify omnidirectional traffic, we avoided, to the best of our visual ability and judgment, labeling stationary bees. In particular, in the above image, all stationary bees on the landing pad are left unlabeled because they do not contribute to traffic.

A random sample of 60 CH=1 videos was drawn from a set of 500 CH=1 videos from apiaries in Logan, Utah (May to September 2020) and Grass Valley, California (June 2022). Another random sample of 60 CH=2 videos was drawn from a different set of 500 CH=2 videos from different apiaries in Logan, Utah (May to September 2019) and Tucson, Arizona (June to September 2021). We used 30 middle images from each of these 120 videos (3600 images in total) as the *evaluation dataset* for the bee object inference experiments with the three trained models. We took a sample of 100 videos (50 CH=1, 50 CH=2) from an apiary in North Logan, Utah (May to September 2018) and an apiary in Tucson, Arizona (July to October 2022). These apiaries were different from the apiaries of the evaluation dataset. We took a random sample of 10 videos (5 CH=1, 5 CH=2) from the sample of 100 videos and used these 10 videos to create a *training dataset* of flying bee images for the models. The 10 videos had 7440 frames (744 frames per video) where we manually labeled 23,173 flying bee objects in 5819 frames with the CV Annotation Tool (CVAT) (www.cvat.ai, accessed on 27 July 2023) (see Figure 4). The bee image dataset curation was done on a Hewlett Packard computer (model Z240, x86_64, i7-6700 CPU at 3.40 GHz, 8 CPUs) running Ubuntu 18.04. In each frame, we labeled only full-size, unoccluded flying bee objects. It should be noted that the YOLO data labeling standards do not require that all objects of a specific class be labeled in each image so long as enough objects of each class (e.g., ≥4000 per category [33]) are labeled in all images.

### 2.3. Bee Object Inference

We trained each model on the training dataset (See Section 2.2) with a random 70/30 train/test split and followed all training recommendations in the documentation from the Darknet public code repository [33]. The main model configuration training parameters are given in Table 2. We set the stopping criterion to the absolute difference between the average losses of two consecutive training runs not exceeding 0.01, where each training run consisted of the number of iterations in the last column of Table 2. We left unchanged the default image augmentation techniques used by the Darknet trainer and did not make any changes in the default activation functions or individual layers of the models. The number of object classes was set to 1 with the label BEE. The synapse weights of each model were persisted every 1000 iterations of a run for robustness to power outages. When the trainer finished a run, the average loss was compared to the average loss of the previous run and, if the absolute difference between the two losses did not exceed 0.01, the persisted model’s synapse weights of the run with the smallest loss (of the two losses) were saved for the subsequent bee object inference evaluation on the evaluation dataset. If the absolute value of the average loss difference exceeded 0.01, the smallest average loss became the previous loss and the persistent model with the smallest average loss was trained for another run. The average loss ties were broken arbitrarily.

The models were trained on the GTX-980 GPU computer (see Figure 2). To minimize power consumption during training, the wireless and wired Internet connections were disabled to prevent background updates, all USB devices were disconnected, and the computer’s monitor was turned off and the keyboard detached, except when we re-launched the trainer from the Ubuntu command line.

### 2.4. Object-Motion Alignment

Since the comparison of different motion detection methods was beyond the scope of our investigation, we used our default motion detection algorithm implemented with OpenCV 2.1 in Python 3.6.7 on Ubuntu 18.04. The method works on two consecutive RGB frames $F_{i}$ and $F_{i+1}$ of a video, and is inspired by the CV methods and techniques in [34]. Both frames are grayscaled and the Gaussian blur with a 5 × 5 kernel is applied to them. The pixelwise absolute difference, i.e., $F_{d}=\left|F_{1}-F_{2}\right|$, is computed between the blurred frames. $F_{d}$ is dilated with a 5 × 5 kernel to make the differences more pronounced for subsequent contour detection. The binary thresholding with a threshold of 20 is applied to $F_{d}$ and the contours are found with chain approximation, i.e., the procedure cv2.findContours() with the keyword parameter method set to cv2.CHAIN_APPROX_SIMPLE. Contours whose area is less than 1000 pixels are discarded, because they are too small to contain one flying bee object. Each thresholded contour is enclosed with the smallest rectangle (x,y,w,h), where (x,y) are the column and row of the top left corner of the rectangle and *w* and *h* are the rectangle’s width and height. Each enclosing rectangle is considered a *motion region* whose center, i.e., (x + w/2,y + h/2), is stored in a kd-tree [35] implemented with the class KDTree in the scipy.spatial Python package for alignment with bee objects inferred by a trained YOLO model in the same frame.

A bee object inferred by a YOLO model is a rectangle $\left(x_{1},y_{1},x_{2},y_{2}\right)$, where $\left(x_{1},y_{1}\right)$ and $\left(x_{2},y_{2}\right)$ are the respective column and row coordinates of the rectangle’s top left and bottom right corners. The score threshold parameter for each YOLO model was set at 0.7. To align inferred bee objects, i.e., YOLO boxes with scores of at least 0.7, with motion regions, the values of $c_{x}=x_{1}+\left(x_{2}-x_{1}\right)/2$ and $c_{y}=y_{2}+\left(y_{2}-y_{1}\right)/2$ were computed for the center of each inferred bee object box, and the kd-tree *T* was queried for the motion region centers within the Euclidean distance of ≤50 pixels to the center of each inferred bee object box. The bee object was considered *motion-aligned* if at least one motion region’s center was found in the kd-tree within the distance threshold, otherwise the bee object was considered *motion-unaligned*. The distance ties were broken arbitrarily.

For every frame, the foregoing object-motion alignment algorithm returned three sets of objects: the set of motion regions, the set of motion-aligned inferred bee objects, and the set of motion-unaligned inferred bee objects. The bee object inference, motion detection, and object-motion alignment algorithms were executed sequentially on each video frame, i.e., object inference, then motion detection, and then object-motion alignment.

### 2.5. Bee Object Inference Accuracy

**Figure 5:**
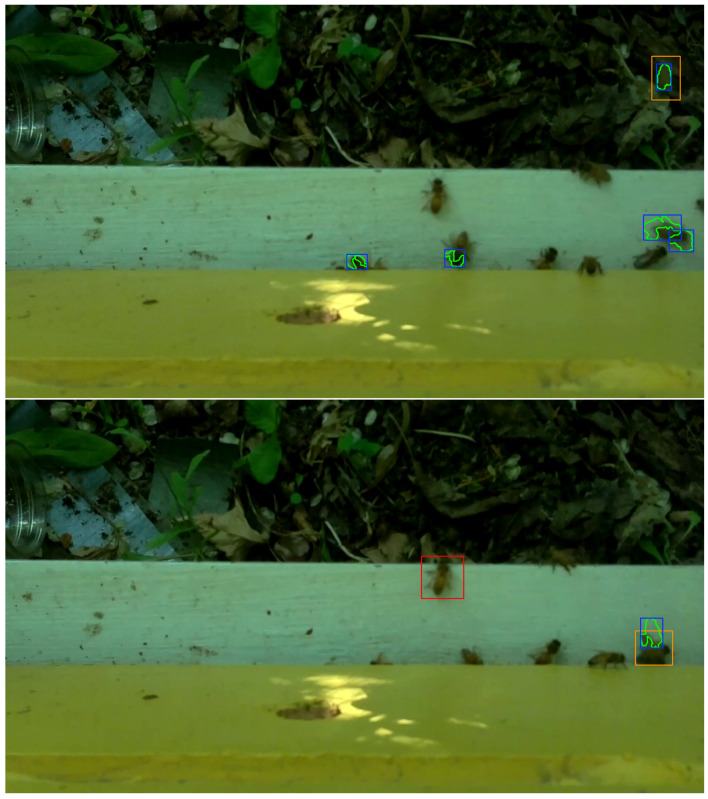
**Top**: Category BTP_BFLMTP (Bee True Positive and Bee Flight Motion True Positive). **Bottom**: Categories BTP_BCRMTP (Bee True Positive and Bee Crawling Motion True Positive) and BTP_BMTN (Bee True Positive and Bee Motion True Negative).

**Figure 6:**
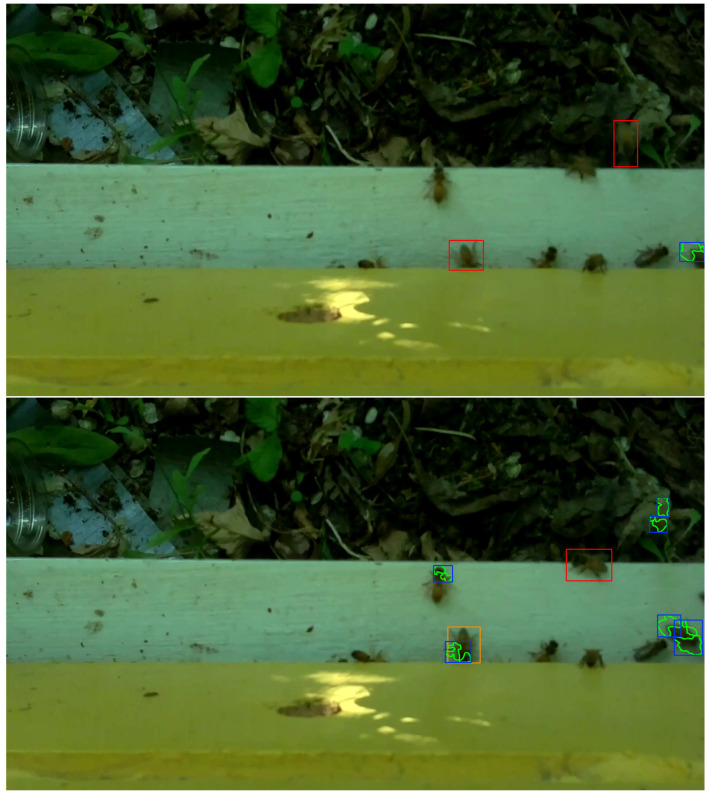
**Top**: categories BTP_BMFN (Bee True Positive and Bee Motion False Negative). **Bottom**: categories BFN_BFLMTP (Bee False Negative and Bee Flight Motion True Positive) and BTP_BCRMTP (Bee True Positive and Bee Crawling Motion True Positive).

**Figure 7:**
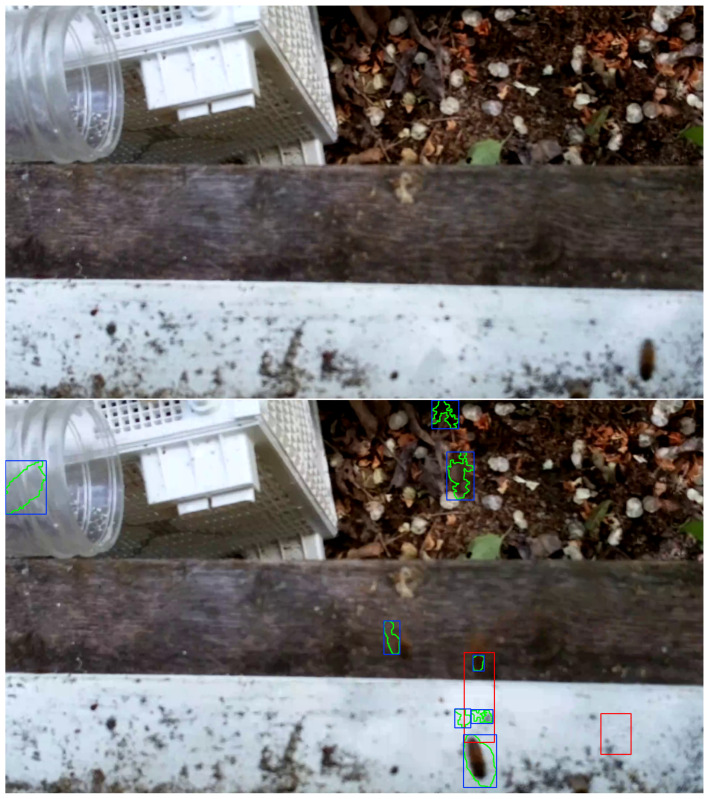
**Top**: category BFN_BFLMFN (Bee False Negative and Bee Flight Motion False Negative). **Bottom**: category BFP (Bee False Positive).

The middle 30 frames, i.e., frames 358 to 387, from each video in the evaluation set of 120 videos (See Section 2.2) were processed with OmniBeeM where bee object inference was done with YOLOv3, YOLOv4-tiny, and YOLOv7-tiny and object-motion alignment was as described in Section 2.4. To make manual accuracy evaluation easier, we wrote procedures to enclose every motion-aligned inferred bee object in an orange rectangle and every motion-unaligned inferred bee object in a red rectangle, where the rectangle coordinates were the coordinates of inferred bee object boxes returned by the trained YOLO models. The motion regions were marked with yellow polygon motion curves enclosed in blue rectangles. Figure 5, Figure 6 and Figure 7 and the concomitant explanations in the text elucidate our color marking scheme. All 30 marked frames from each evaluation video were saved on the GPU computer’s hard drive. We then manually evaluated the bee object inference accuracy in each of the 3600 marked frames, i.e., 1800 CH=1 frames and 1800 CH=2 frames.

Our evaluation of bee object inference accuracy involved seven categories designed for this study. The first category, BTP_BFLMTP, abbreviates the phrase *bee true positive and bee flight motion true positive* and describes the situation when the object inference model accurately infers a bee object and there is a motion region that satisfies two criteria: (**C1**) it is sufficiently close to the inferred bee object (this criterion is controlled by the threshold of ≤50 pixels in the kd-tree queries) and (**C2**) it has a visually detectable intersection with the inferred bee object. The former criterion is detected programmatically, while the latter is detected during the manual evaluation by a human evaluator. The upper image in Figure 5 illustrates this category: an orange rectangle in the upper right corner of the image is a motion-aligned inferred bee object. It is motion-aligned because, inside the orange rectangle, there is a blue rectangle enclosing a motion region marked by a yellow polygon curve connecting individual pixels of a flying bee object. The category describes an ideal case where an inferred object contains a motion region (or vice versa) or when there is a significant, visually detectable overlap between the two regions.

The second category, BTP_BCRMTP, abbreviates the phrase *bee true positive and bee crawling motion true positive* and describes the situation when the object inference model accurately infers a bee object and there is a motion region that satisfies **C1** and **C2**, but the bee object is not in flight but crawling. We checked the latter condition by looking at the bee object in the previous frame to determine if there was, indeed, a crawling motion. If the inferred bee barely moved or moved its wings or slightly changed its orientation, then we put the motion-aligned inferred bee object in this category. The lower image in Figure 5 illustrates this situation: an orange rectangle in the lower right part of the image, on the horizontal intersection line between the white landing pad and the yellow hive super on top of the pad, is an inferred bee object. This region overlaps with a blue motion region, but this bee object barely moved when its position in this frame was compared to its position in the previous frame.

The third category, BTP_BMTN, abbreviates the phrase *bee true positive and bee motion true negative* and describes the situation when the object inference model accurately infers a bee object, but the inferred bee object is stationary, i.e., in the human evaluator’s judgment, its position in the current frame is the same as in the previous one, and no motion region is detected that satisfies **C1** and **C2**. Hence, the second half of the phrase — *bee motion true negative*. This category is illustrated with a red rectangle drawn around a stationary bee slightly right of the center of the white landing pad in the lower image of Figure 5. Since this inferred bee object made no motion from its position in the previous frame, we counted this bee object as an instance of BTP_BMTN.

The fourth category, BTP_BMFN, abbreviates the phrase *bee true positive and bee motion false negative* and describes the situation when the object inference model accurately infers a bee object, but no motion region is found satisfying **C1** and **C2**. The upper image in Figure 6 illustrates an instance of BTP_BMFN with a red rectangle enclosing a correctly inferred flying bee object on the right side of the image above the white landing pad. However, there is no motion region detected nearby that satisfies **C1** and **C2**.

The fifth category, BFN_BFLMTP, abbreviates the phrase *bee false negative and bee flight motion true positive* and describes the situation when the motion detection algorithm accurately identifies a bee object in flight but the object inference model fails to infer a bee object in such a way that **C1** and **C2** are satisfied. The lower image of Figure 6 gives an example of this category with two accurately detected blue-yellow motion regions above and to the right of a correctly inferred motionless bee inside a red rectangle right on the edge of the white landing pad. The motion regions are correctly detected around the thorax and body of a flying bee object, yet the object inference model (YOLOv4-tiny in this case) failed to infer a bee object in the vicinity of either motion region. Incidentally, the lower image of Figure 6 contains an instance of the previously discussed category BTP_BCRMTP with an orange rectangle visibly overlapping a blue-yellow motion region slightly right of the middle of the white landing pad.

The sixth category, BFN_BFLMFN, abbreviates the phrase *bee false negative and bee flight motion false negative* and describes the situation when a human evaluator identifies a bee object in flight, but neither the object inference model nor the motion detector recognize anything in the region of the image with the bee object. The upper image of Figure 7 illustrates this category: a flying bee in the bottom right corner of the image above the landing pad is not detected by the object inference model or the motion detector.

The seventh category, BFP, abbreviates the phrase *bee false positive* and describes the situation when the object inference model infers a bee object where there is no bee object regardless of nearby motion regions or lack thereof. The lower image of Figure 7 illustrates this category with a red rectangle on the right side of the white landing pad signaling a false inference of a bee object.

For each model, we counted the instances of the first four categories as true positives (TP), i.e., instances where the model accurately inferred the presence of a bee object irrespective of motion. The instances of the fifth and sixth categories were counted as false negatives (FN), i.e., the model’s failures to infer true bee objects. The last category gave us the counts of false positives (FP) where the model inferred the presence of a bee object where there was none. We used four standard metrics, i.e., Precision, Recall, F1, and Intersection Over Union (IOU) in (1), to compare the performance of the models.

(1) $$\begin{array}{ccc} Precision & = & \frac{TP}{TP+FN}\\ Recall & = & \frac{TP}{TP+FP}\\ F1 & = & 2\frac{Rrecision\times Recall}{Precision+Recall}\\ IOU & = & \frac{TP}{TP+FP+FN} \end{array}$$

### 2.6. Energy Efficacy and Operational Energy Footprint

**Figure 8:**
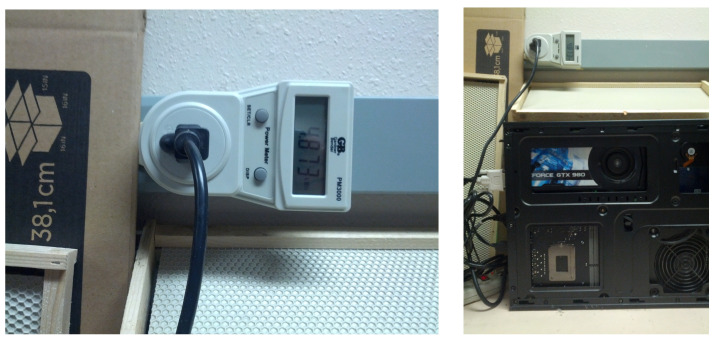
**Left**: A Gardner Bender(TM) PM3000 power meter plugged into a wall outlet. **Right**: A GEFORCE GTX-980 GPU computer plugged into a PM3000 power meter.

We recorded the total physical time in hours to curate the training dataset and the total power amount to complete the curation. The power amount was recorded with a Gardner Bender(TM) PM3000 Power Meter (see the left image in Figure 8). The meter was reset before each video labeling session and the total cumulative power amount (CPA) in kilowatt-hours (kW-h) on the meter’s display was logged at the end of the session.

The physical run time in hours was programmatically logged for each training run of each model, excluding power outage periods. The training energy consumption data were taken from another PM3000 meter plugged into an electrical wall outlet and the GPU computer was plugged into the meter, as shown in the right image of Figure 8. We reset the meter prior to each new training run. When the training was finished, the total CPA in kW-h on the meter’s display was added to the CPA of the previous runs. The power use rate of training each model was estimated as the CPA divided by the total number of hours to train the model until the stopping criterion was satisfied.

For each model, we recorded the total power amount spent on making the model operational, i.e., data curation, model training, and manual evaluation. We refer to this energy amount as the model’s *data engineering energy footprint*. We measured the energy efficacy (EFF) of each model by the formula in (2) that we designed for this investigation, where the parameter *M* is the value of a specific performance metric of the model (e.g., F1), *A* is the total data engineering energy footprint of the model, and λ is a scaling factor which we set to $10^{2}$. This formula is designed to estimate the number of performance metric units per every unit of the model’s data engineering energy footprint.

(2) $$\begin{array}{ccc} EFF(M,A) & = & \lambda \frac{M}{A} \end{array}$$

The energy lifespan of a DL or ML model is not limited to the energy footprint incurred during the data engineering phase. Once trained and evaluated, the model is integrated into a data pipeline and contributes to the energy footprint of the pipeline, which we refer to as the *operational energy footprint* (OEF). To this end, we calculated the monthly and seasonal energy footprints of BeePiP with each trained and evaluated YOLO model in Logan, Utah. BeePiP consisted of 10 video loggers and one GEFORCE GXT-980 GPU computer.

## 3. Results

It took us 220.33 h to label 23,173 flying bee objects in 5819 frames of the 7440 frames of the 10 training videos. The total energy footprint of labeling bees in the videos was 11.6 kW-h. The mean average bee labeling rate was 105.17 bees per hour. The training times and energy footprints of the models are given in Table 3. It took us an additional 180.41 h to evaluate the trained models on the 120 CH=1 and CH=2 evaluation videos with the seven categories described in Section 2.5. We did this evaluation on the same computer where we did the image labeling and incurred an additional energy footprint of 9.56 kW-h. Table 4 details the obtained evaluation results.

Excluding the time spent on algorithm design, software engineering, and hardware assembly and testing, the total amount of physical time it took us to train and evaluate the models was 1864.64 h: 220.23 h of manual image labeling, 1464 h of model training, and 180.41 h of manual evaluation of the model’s performance on the evaluation videos. The total CPA for making the models operational was 351.55 kW-h: 11.60 kW-h for image labeling, 300.39 kW-h for model training, and 9.56 kW-h for model evaluation. Table 5 summarizes the data engineering energy footprints of each model. The models’ efficacy measures are detailed in Table 6.

Table 7 gives the video processing time and power use rates and monthly power amounts of the trained models on the GPU computer (see Figure 2 and Figure 8). Table 8 presents monthly and seasonal energy footprints estimates (assuming that each month has 24×7×4=672 h) of BeePiP 10 on-hive video loggers and one GEFORCE GTX-980 computer in Logan, UT, USA.

## 4. Discussion

### 4.1. Accuracy

Since it is not feasible to train DL or ML bee object inference models for each specific apiary, we deliberately chose the training and evaluation videos not only from different apiaries, but also from different years and different bee races in order to a) make our evaluation imitate real-world situations and b) test the generalization capacity of each model. Table 3 shows that YOLOv3 took much longer to train (720 h) than YOLOv4-tiny (552 h) or YOLOv7-tiny (192 h) and had a significantly higher training energy footprint (155.89 kW-h) than YOLOv4-tiny (119.51 kW-h) or YOLOv7-tiny (24.99 kW-h). The performance accuracy statistics in Table 4 indicate that YOLOv3 was better at generalization than the other two models in that its Precision, Recall, F1, and IOU were higher.

YOLOv3 can be used to quantify omnidirectional traffic in videos from on-hive loggers, because it had no false positives in the CH=1 or CH=2 videos of the evaluation set. However, it can be used to estimate only lower bounds due to false negatives (581 on CH=1 videos and 2577 on CH=2 videos) and, consequently, can be expected to underestimate the true amount of omnidirectional traffic. Like YOLOv3, YOLOv4-tiny had no false positives on the CH=1 videos. However, it had more false negatives than YOLOv3 on the CH=1 videos (748 vs. 581) and more false positives on the CH=2 videos (24 vs. 0). The Precision, F1, and IOU of YOLOv7-tiny were lower than YOLOv3. However, YOLOv7-tiny had higher Precision, F1, and IOU on the CH=1 videos than YOLOv4-tiny and achieved comparable values in these performance metrics with YOLOv4-tiny on the CH=2 videos. The Recall of YOLOv7-tiny was lower than the Recall of YOLOv4-tiny on the CH=1 videos (0.47 vs. 1.00) and the CH=2 videos (0.28 vs. 0.90) due to higher counts of false positives: 300 vs. 0 in CH=1 and 498 vs. 24 in CH=2. While YOLOv4-tiny and YOLOv7-tiny also had false positives, their counts were considerably smaller than the counts of false negatives. Thus, like YOLOv3, YOLOv4-tiny and YOLOv7-tiny are also expected to underestimate the true amount of omnidirectional traffic due to the large numbers of false negatives in the evaluation videos.

In viewing multiple bee traffic videos, we noticed four recurrent flight patterns which we called (1) *straight*, (2) *inward/outward zigzag*, (3) *land-and-crawl*, and (4) *parallel*. The *straight* pattern is characterized by bees, mostly foragers, flying in and out of a hive along more or less straight trajectories. The bees that use this trajectory fly at higher speeds. Our hypothesis is that these are experienced foragers that are confident about their trajectories, because they know their routes. We also noticed a few drones using this type of trajectory. The second pattern is characterized by foragers that fly toward the hive, then fly away from the hive, then fly forward or parallel to the hive, and then fly in or away. We called this flight pattern *inward zigzag*, because the bee initially flies toward the hive. In case, the bee flies away at the end of the the pattern, it may be a scout bee from a different colony. The *outward zigzag* pattern is seen in foragers flying out of the hive. In this pattern, a forager flies out, turns around in the air, flies toward the hive, and then turns around and flies away. Our conjecture is that this pattern characterizes bees from the hive that may not be familiar with their routes and must orient themselves before flying in. The *land-and-crawl* pattern characterizes a bee that follows a straight or inward zigzag trajectory and then lands on the landing pad or near the landing pad and then crawls into the hive or stays in place without any visible motion. We have no hypothesis of the bees that exhibit this pattern. The *parallel* pattern shows in bees that fly more or less parallel to the landing pad for the duration of the video. Our hypothesis is that it is either this pattern characterizes orienting bees from the monitored hives or bees from other colonies. The OmniBeeM method does not track trajectories. Trajectory tracking may be feasible in low-traffic videos when a couple of bees are flying in a video. In medium- or higher-traffic videos, it may not be feasible due to criss-crossing and overlapping trajectories unless individual bees are tagged (e.g., [7]), which, by definition, makes the method invasive.

The OmniBeeM method counts all flying bee objects in a frame and uses this as a numerical measurement of traffic. The same bee, so long as it remains in flight, can be counted in different frames of the same video, which may overestimate the overall count of flying bees. It is unclear to us, however, whether this overestimation, if present and consistent, interferes with the assessment of long-term traffic trends. Our results show that the three models misclassify as flying some crawling and stationary bees. This limitation can be addressed by (1) enlarging our image dataset with more flying bee objects and (2) excluding all inferred bee objects detected on the landing pad.

If the research hypothesis by Marceau et al. [1] is correct, i.e., bee traffic at the hive’s entrance is a contributing factor to the hive’s productivity and health, then it makes sense to use computer vision traffic quantification methods to assess bee colony strength and, hence, the price paid per pollination colony. Winfree et al. [36] report that the flowers of watermelon and muskmelon are active for only one day when they open at daybreak and close by early afternoon. If this day is known in advance, then computer vision traffic quantification can be used to choose on the basis of the previous traffic assessments which colonies can be sent to a specific pollination event.

### 4.2. Energy

The energy footprint of making DL and ML models operational is not confined to model training. It also includes the footprints of data curation and model evaluation. As shown in Table 3, the model training alone took 1464 h and 300.39 kW-h. To put this energy footprint in perspective, we note that, according to the energy bills available to us from the city of Logan, Utah, a 3-bedroom apartment with four residents in Logan, Utah used 382 kW-h in November 2017 and 491 kW-h in December 2019. Thus, training the three models for this investigation was equivalent to 79% of the apartment’s total energy amount in November 2017 and 61% of its energy amount in December 2019. If we look at the monthly and seasonal OEF estimates in Table 8, we can see that the seasonal OEF of BeePiP with YOLOv3 in Logan, Utah is 807.60 kW-h, which is 93% of the apartment’s 2-month total CPA (873 kW-h). The seasonal energy footprints of BeePiP with YOLOv4-tiny and of BeePiP with YOLOv7-tiny are, respectively, 89% and 81% of the same 2-month total. These percentages are in line with the energy footprint estimates of DL models by other researchers, e.g., training a single DL model can emit over 626,000 pounds of carbon dioxide, i.e., the amount equivalent to the carbon dioxide emissions of five U.S. automobiles over their lifespans [20]. Cloud computing takes vital resources such as water and energy from the environment and puts back into it potentially harmful by-products such as heat, carbon dioxide, and electromagnetic radiation.

Since many DL models have significant data engineering energy footprints, we need a means to estimate their energy efficacy (EFF). We addressed this question by designing an energy efficacy formula in (2) to estimate the number of performance accuracy units per unit of the data engineering energy footprint. From the perspective of accuracy, YOLOv3 was better than YOLOv4-tiny and YOLOv7-tiny. However, from the perspective of EFF in Table 6, YOLOv7-tiny was better than either YOLOv3 or YOLOv4-tiny. Furthermore, YOLOv7-tiny could infer bee objects in individual images, albeit insufficiently fast, on a single Raspberry Pi computer, which, in principle, makes it feasible to achieve faster run times and lower OEFs by distributing bee object inference with YOLOv7-tiny across several Pi computers. Thus, YOLOv7-tiny appears to us to be the most promising model to train on larger datasets if a primary objective is to obtain non-invasive computer vision traffic quantification methods with maximal EFFs and minimal OEFs.

### 4.3. Related Bee Traffic Research

Table 9 lists several research projects related to our investigation. Kimura et al. [6] proposed a method to count bees and measure their motion on internal frames of an observation hive with transparent walls. The system recorded videos with a digital camera (GR-HD1; JVC, Yokohama, Japan) mounted on the side of the hive. The camera had a resolution of 720 × 480 pixels and a frame rate of 29.97 fps. The size of an inside hive frame was 44 cm in width and 19.6 cm in height. The proposed method included three steps: the extraction of a honeybee-code image from a whole image of the inside frame, the separation of single and plural regions of bees from the code image using an average honeybee body size and shape given as parameters, and tracking of bee motions in sequential images. The vector quantization method separated a single image into multiple regions with approximately the same characteristics. Each such region was represented by a centroid vector, also called the *code vector*. The researchers experimentally determined that eight code vector values were optimal to separate individual objects on one observation hive frame. These code vectors corresponded to the bee body, the dark and light of the bee wing, the hive, the hive frame, and the background and noise. Eight code vectors represented all identifiable objects in four-dimensional space. The method was evaluated on three randomly selected frames from a 10-second movie (300 frames) and the percentage of the objects correctly identified by the system in each frame was manually counted. The vector quantization method identified 510 bees out of 704 manually identified bees in the first frame, 522 out of 718 in the second frame, and 516 out of 700 in the third frame, which translated to an average accuracy of 72.95%. The method also determined the active areas in the bee frame by extracting the trajectories of walking bees. Some data on the honey bee waggle dance were obtained.

Chen et al. [7] proposed a system for counting tagged bees and identifying their orientation at the hive’s entrance. The system’s hardware included an infrared camera (DMK31AU03, The Imaging Source Europe GmbH, Germany ) and an infrared LED light source for lighting stabilization. The camera was connected via a USB port to an Intel Core 2 Duo 2.1 GHz Windows workstation with 4 GB of RAM. The direction and speed of bee motion was restricted with a passage on the landing pad constructed with 3 mm transparent acrylic sheets. The external dimensions of the passage were 145 mm × 80 mm × 9 mm (length × width × height). The camera was positioned above the passageway to acquire images of bees crawling in and out. The exact hive type was not specified. To identify foragers in videos, small circular paper tags were glued to the backs of 100 bees. Each tag had a diameter of 3 mm and several special characters to denote identification and orientation. To be tagged, each bee was immobilized by being placed in a freezer for 3–4 min. The fine hairs on the back of the immobilized bee were removed and an instant adhesive was used to glue the tag to the bee’s thorax. To locate tagged bees in a video frame, a circular Hough transform [37] was used to detect the presence of circles. A black positioning dot character on the tag was used to identify the orientation of the characters. The character symbols were segmented with an optical character recognition package, and a support vector machine (SVM) classifier was trained to recognize individual characters, thereby identifying the individuality of the bee and its orientation. The character symbol recognition and identification accuracy rates were 98% and 86%, respectively. The system’s performance was tested in a laboratory by placing five tagged bees in the closed passageway where the bees walked back and forth searching for the exit. The camera was taking images for 30 min and correctly identified 154 entry and exit instances out of 189 ground truth instances. The system was deployed for 15 days in the field to track 100 tagged foragers. Due to forager attrition, 82 bees were detected on day 1; 59—on day 2; 40—on day 3; by day 8, the number of detected bees fell to 4.

Dussaubat et al. [8] proposed an optic bee counter system that included a digital camera facing down the entrance of a 4-frame nuc hive. Eight tunnel passages were placed at the bottom of the entrance. Each tunnel allowed only one bee to enter or exit the hive in such a way that the bee’s thorax was exposed to the camera. The width of the passages corresponded to the camera’s lens angle. The camera’s model, image resolution, and frame rate were not specified. The only performance characteristic in the publication was that the system’s bee counting software achieved a minimal error of 3–4% when counting bees in the tunnels. The software controlled three cameras, one camera per each 4-frame nuc to analyze the images in real time. The frame rate was dynamically adjusted to minimize the chance of missing bees in the tunnels.

Chiron et al. [9] proposed a system to detect and track honey bees in the hive’s vicinity with 3D stereo vision methods. The image acquisition was done with a G3 EV Stereo Camera (TYZX, Menlo Park, CA, USA). The camera had a resolution of 752 × 480 pixels and a frame rate of 50 fps and was mounted on a 2-super Langstroth hive facing down to the landing pad. No hive modifications were done. The camera generated pairs of left and right grayscale images and a corresponding disparity map. A stereo pair matching algorithm computed *holes*, i.e., unmatched areas, for which there was no certainty that they corresponded to flying bee objects. Flying bee objects were identified by peaks on the depth map or, under certain conditions, by holes. For multi-target tracking, a method was proposed based on the Kalman filter [38] and the Global Nearest Neighbors (GNN), [39] whereby each flying bee target was associated with a Kalman filter and the GNN matched uncertainty measurements against known tracks. The software and hardware details were not specified. The evaluation dataset consisted of 500 randomly selected frames for segmentation ground truth under different lighting conditions and 80 manually annotated trajectories in ≈1000 frames from one Langstroth hive. 4.8% of bees were incorrectly marked due to human error. The evaluation of the segmentation method resulted in 4.15% of false negatives and 19.54% of false positives. The tracking performance decreased with the number of tracked bee targets with approximately 82% of recovered targets under normal conditions.

**Table 9:** Related bee traffic research; NA—not available in the text of the publication or supplementary materials; HWR—hardware; SWR—software; DC—digital camera; IC—infrared camera; SC—stereo camera; HDC—high definition camera; OBS—observation; UNSPC – unspecified; 4FN—4-frame nuc hive; MFD—modified hive; UMFD—unmodified hive; 1S—one super; 2S—two super; 3S—three super; LST—Langstroth; RPi—Raspberry Pi.

Cite	Objective	HWR	SWR	Data	Hive
[6]	Counts of walking	DC	NA	3 images from	OBS
	bees on inside			1 10-second	
	frames; walking bee			video from	
	trajectory detection			1 hive	
[7]	Tagged forager	IC	C++,	NA	UNSPC;
	counts at hive	LED Light	OpenCV		MFD
	entrance				
[9]	Counts of flying	SC	NA	1500 images	2S LST,
	bees; flight trajectory			from 1 hive	UMFD
	tracking in hive				
	vicinity				
[8]	Counts of walking	DC	NA	NA	4FN,
	bees at hive				MFD
	entrance				
[12]	Bee counts;	RPi B+,	C++,	400 images	1S LST,
	in-out traffic	RPi Cam	OpenCV,	from 1 hive	MFD
	measurement at		MySQL		
	hive entrance				
[11]	Pollen bearing	RPi 2,	Python,	150 images	NA;
	forager counts	RPi 2 HDC,	OpenCV,	from 1 hive	MFD
	at hive entrance	Android phone	MATLAB		
[13]	Pollen bearing	DC,	NA	3500 images	4FN,
	forager counts	Jetson TX2,		from 5 nucs	MFD
	at hive	LED Light,			
	entrance	2 RXT 2080 GPU			
[15]	Directional bee	RPi 2,	C, Python,	23,808 images	1S/2S
	traffic estimation	RPi DC	OpenCV	from 2 hives	LST,
	in hive vicinity	RPi DC			UMFD
[40]	Bee counts	RPi 2,	Python,	54,678 images	1S/2S/3S
	in hive vicinity	RPi DC	OpenCV	from 5 hives	LST,
					UMFD
This study	Counts of flying	RPi 2,	C, Python,	9419 images	1S/2S
	bees in hive	RPi DC,	Darknet,	from 10 hives	LST,
	vicinity	GTX-980 GPU	OpenCV		UMFD

Babic et al. [11] proposed a pollen-bearing forager counting system that consisted of a Raspberry Pi 2 computer coupled to a Raspberry Pi High-Definition (HD) camera with a resolution of 1280 × 720 pixels and a frame rate of 30 fps. The system included an Android cell phone for a wireless area network (WLAN) connection to an Intell i3 workstation with 8 GB of RAM. Background subtraction was applied to each video frame with the Mixture of Gaussians (MOG) [41]. The Pi camera faced down on the landing pad. A glass plate was placed 2 cm above the pad, thus forcing the bees to crawl ≈11 cm before entering or leaving the hive. For each non-background blob with specific size criteria, a four-feature vector descriptor of color variance and eccentricity coefficients was computed. A two-way nearest means classification (pollen-bearing forager vs. non-pollen-bearing forager) of descriptors was done to obtain the counts of pollen bearing workers. This classification was executed on the Raspberry Pi computer. The images were also sent to the workstation where they were processed by an SVM. The dataset consisted of 50 RGB images of pollen-bearing foragers and 50 RGB images of foragers without pollen loads taken from 40 RGB video frames from a managed hive of unspecified type. The testing was done on 50 video frames with a total of 354 bees from the same hive. The two-way blob classification method achieved an accuracy of 88.7% and the SVM classifier achieved an accuracy of 87.42%.

Tu et al. [12] proposed a system for counting bees at the hive’s entrance and measuring incoming and outgoing traffic. The system’s hardware consisted of an Internet-enabled Raspberry Pi B+ computer coupled to a Raspberry Pi camera placed in a box with stable LED light sources with diffusers and a mirror for controlling lighting conditions. The camera was directed toward the mirror placed at a 45-degree angle to view the bees from below. The camera had a resolution of 1920 × 1080 pixels and a frame rate of 25 fps. Videos were captured in H.264 format and converted to MP4. RGB frames were extracted from MP4 videos. The bee counts and estimates of in-out traffic were calculated from individual frames. The bees were allowed to enter or exit the hive only through a special passage in order to probit the overlapping of individual bees. Each video image was grayscaled and the mean overall pixel intensities were calculated. A threshold was chosen on the basis of the computed mean to segment the image. If the total number of foreground pixels obtained through background subtraction in the binary image was greater than a manually determined threshold, the counting of bees was calculated by using a manually crafted linear regression equation as a function of the number of bees in the image, the total number of foreground pixels, and two manually chosen linear regression parameters. Inferential statistics were used to estimate the orientation of bees and measure incoming and outgoing traffic. The computation ran on the Raspberry Pi computer and the extracted bee counts and in-and-out traffic evaluations were sent to an off-board MySQL database on an unspecified Internet host. The H.264 and MP4 videos were deleted. The dataset consisted of 25 training and 100 testing 30-second videos from a hive with one deep super with 6 to 25 honey bees in each frame recorded at 30-minute intervals. To make image processing run on the Raspberry Pi, the frame rate was reduced to 5 fps, and the videos were taken every 10 min. To evaluate the system’s performance, 400 images were randomly selected from the same hive and all bees were manually counted. One video from the same hive was used to evaluate the in-out traffic measurements. The R2 coefficient between the system’s counts and manual counts was 0.987.

Ngo et al. [13] designed and deployed a pollen-bearing forager counting system for 4-frame nuc hives. The system used an off-the-shelf RGB web camera (Model: C920, Logitech International S.A., Switzerland). The camera had an image resolution of 640 × 480 pixels and was coupled to an embedded Jetson TX2 system via a USB port. The camera was enclosed in a black acrylic box equipped with a red LED panel for light control. The average frame rate was 25 fps. The camera looked down at the landing pad. A transparent pathway was placed on the landing pad, which forced the bees to walk an unspecified distance in and out of the hive. The YOLOv3-tiny model was trained on two GEFORCE RTX 2080 GPUs with 8 GB of RAM to do the two-way classification of pollen-bearing vs. non-pollen-bearing foragers. Images were extracted from the video stream of bees walking in and out. The trained YOLOv3-tiny model in conjunction with the Kalman filter and the Hungarian algorithm returned the counts of in-coming pollen-bearing, incoming non-pollen-bearing and outgoing non-pollen-bearing bees. The training dataset included 3000 images and the testing dataset included 500 images from five 4-frame nuc hives. The mannual image annotation was done with LabelImg [42]. The training of YOLOv3 was done for 20,000 iterations with a learning rate of 0.001 until the average loss was under 0.17. The performance of the system was evaluated on 34 10-minute randomly selected videos at different times and different nuc hives. In each video, the number of incoming pollen-bearing bees was manually counted and compared to the system’s counts. The number of pollen-bearing bees in each video ranged from 34 to 166. The mean absolute error regression analysis of manual and automatic counts showed that the system slightly underestimated the manual counts.

Kulyukin et al. [15] proposed BeePIV, an algorithm that uses particle image velocimetry methods [16] to estimate the amount of incoming, outgoing, and lateral bee traffic in videos from on-hive loggers. BeePIV converts video frames to particle motion frames, assembles motion points into motion particle clusters, and computes particle displacement vector fields. Individual displacement vectors are classified as incoming, outgoing, and lateral, and the vector counts in each class are used as measurements of incoming, outgoing, and lateral traffic. The system’s hardware consisted of a Raspberry Pi B 1.2 computer with four cores coupled to an 8-megapixel Pi camera. The hardware units were packaged in a standard Langstroth super and placed on top of a standard Langstroth hive with the camera looking down on the landing pad. No structural modification of the hive was done. Raw H.264 30-second videos with a resolution of 1920 × 1080 pixels and a frame rate of 25 fps are captured every 15 min. Each extracted frame was converted to PNG and resized to the 60 × 80 resolution for *in situ* processing on the Raspberry Pi computer. The mean video processing time was 2.15 min with a standard deviation of 1.03. The evaluation dataset consisted of 32 30-second videos (744 frames per video) from two on-hive loggers at an apiary in Logan, Utah, USA deployed from May to November 2018. The error rate was computed as the absolute difference between the system’s omnidirectional bee count estimates and the human counts varied from 0 to 13 per video with a mean of 3.4 and a standard deviation of 3.97. Obtaining ground truth from human evaluators for directional bee traffic measurements turned out to be humanly impossible due to very large numbers of bee motion vectors detected in high-traffic videos. A mediate method was chosen instead that matched the time series of incoming and outgoing motion vector counts from two hives under different conditions with dynamic time warping (DTW). For hive 1, the mean DTW was 6.08 with a standard deviation of 1.67; for hive 2, the mean DTW was 5.4 with a standard deviation of 1.81.

Kulyukin et al. [40] compared the performance of shallow convolutional networks (SCNs) and reinforced random forests (RRF) as classification methods to determine if image regions selected by motion detection contained bees or bee shadows on four image datasets (BEE1, BEE2, BEE3, BEE4 [43]) of 54,678 honey bee images. For a given dataset, a convolutional network was considered *shallow* if its memory footprint on disk was less than or equal to the footprint of the largest RRF trained on the same dataset, which was 50 MB. The performance of SCNs and RRFs were comparable with the validation accuracies ranging from 88% to 97%.

### 4.4. Perspectives

Of ten projects in Table 9 five (i.e., [7,8,11,12,13]) restricted hive access and one (i.e., [6]) used an observation hive with transparent walls. In addition to restricting hive access, Chen et al. [7] also placed visual paper tags on individual bees. Thus, 60% of the investigations were performed in hive environments never encountered by commercial beekeeping operations or most amateur and professional apiarists who use regular, structurally unmodified Langstroth hives or close variants thereof, such as Dadant [44]. While the results obtained with modified hives are valuable, they will unlikely generalize to the environments encountered by most apiarists. Hive access modifications necessarily interfere with forager flight patterns, because the *land-and-crawl* pattern investigated in restricted hive access studies is only one of the exit and entry patterns of honey bees in managed hives, as we noted in Section 4.1. The two most common patterns in our videos were bees flying in or out at high speeds along straight lines, i.e., the *straight* pattern, or zigzagging in or out at lower speeds, i.e., *inward/outward zigzag*. In both patterns, bees flew in or out without landing or walking on the pad. Thus, restricting hive access with acrylic sheets or similar modifications prevents bees from engaging in these flight patterns.

Many commercial beekeeping operations are migratory. They make their profit through pollination contracts in various far-flung locations, which require them to move large numbers of hives from location to location to keep up with tight pollination schedules. For example, in the U.S., the honey bee pollination colonies are scheduled to be in California in February, in Florida in March, in New York in May, and then in Maine [45]. The February pollination of California almonds requires 2.4 million honey bee colonies, i.e., over three-quarters of the U.S. colonies, most of which are moved there by truck every year [46]. To convince commercial operations to become stakeholders in and adopters of computer vision hive monitoring technologies, the latter must be easily installable, quickly movable from hive to hive and from apiary to apiary, and require zero calibration. These requirements cannot be satisfied if every hive must be modified for access and stable illumination for the system to become operational.

It is understandable why 4-frame nuc hives are used in some experiments. These hives contain only four frames of bees and are easier to mount various hardware components on in order to control the lighting conditions as well as the speed and level of bee traffic. However, the levels of bee traffic in such environments are significantly lower than the bee traffic levels in managed bee colonies in regular hives. At the height of the summer season, a healthy bee colony in a Langstroth hive with 3 or 4 10-frame deep supers has 30 or 40 (instead of 4) full frames and houses ≈ 60,000 bees (Ch. 2, p. 23 in [47]) While the forager numbers fluctuate during the season and depend, among other things, on the queen laying patterns (Part 1, p. 30 in [48]), Tauz (Ch. 3, p. 68 in [49]) estimates that a single colony fields between 100,000 and 200,000 foragers per year, with a single forager doing between 3 and 10 flights per day. Furthermore, Tauz estimates that during the summer, 100,000 foragers complete several million foraging flights (Ch. 8, p. 217 [49]). These numbers indicate that a computer vision system that monitors a regular managed bee hive with unrestricted hive access has to cope with bee traffic levels that are orders of magnitude higher than those of hives (e.g., 4-frame nucs or one-super hives) with restricted access.

None of the related studies in Table 9 did energy efficiency analyses of their systems. Yet, many computer vision models have significant data engineering and operational energy footprints (See, e.g., Table 5 and Table 8), which must be taken into account to attract potential stakeholders. Given the rising costs of electricity in many parts of the world, including the U.S. West [21], the accuracy levels of some DL models may not be affordable to stakeholders. In this regard, it is noteworthy and hopeful, that of 10 studies in Table 9, five advocated for the use of low-power platforms and cameras and, as a consequence, computational methods that do not include DL. It is also noteworthy that several investigations relate estimates of bee traffic to other biotic and abiotic factors such as *Nosema* [8], collected pollen amounts [13], and hive weight [32]. Such relations may warrant further investigation.

Running DL models on the Raspberry Pi platforms continues to be a challenge. While working on this investigation, we successfully compiled and built YOLOv3, YOLOv3-tiny, and YOLOv7-tiny from the C source with the Gnu C/C++ compiler (GCC) on the Raspberry Pi 3 and Pi 4 ARM platforms with the Raspbian OS. However, we were unable to run YOLOv3 with the Darknet detector on individual 1080 × 1980 PNG images either on Pi 3 or Pi 4 even after increasing the pagination limit to 1 GB. While both YOLOv4-tiny and YOLOv7-tiny detected bees on a small set of individual 1080 × 1980 PNG images on both Pi 3 and Pi 4, it took both models, on average, over 2 min to process one PNG frame.

The above observations indicate that the use of these models in hive monitoring data pipelines like Beemon or BeePiP necessitates access to a GPU computer via a local network or to a GPU farm via cloud computing. The latter is problematic, because it incurs financial, ecological, and environmental costs. Whether GPU computers in nearby buildings increase levels of ambient electromagnetic radiation to cause negative impacts on the monitored colonies remains to be seen. A better long-term solution for continuous hive monitoring data pipelines may be to make all computation run *in situ* on low-energy embedded platforms like Raspberry Pi, thus eliminating offboard GPU computing altogether.

## 5. Conclusions

YOLOv3 can be used to quantify omnidirectional bee traffic in videos from on-hive loggers, because it had no false positives and was the best model in terms of Precision, Recall, F1, and IOU. However, this model will likely underestimate omnidirectional traffic due to false negatives.

The performance statistics of YOLOv7-tiny, except for Recall, were better than those of YOLOv3-tiny. In terms of energy efficacy, YOLOv7-tiny was better than the other two models, which indicates that, of the three models, it is the model worth training on larger flying *Apis mellifera* object datasets if a primary objective is to maximize energy efficacy. YOLOv7-tiny had the smallest operational energy footprint after being integrated into a hive monitoring data pipeline of 10 on-hive loggers and one GPU computer. Whether YOLOv7-tiny can eventually be used as a reliable model to quantify omnidirectional traffic in videos from on-hive loggers depends on whether the model’s false positives can be reduced or eliminated and on whether the model’s bee object inference can be distributed across several Pi computers to achieve smaller operational energy footprints.

While DL models have the potential to transform the video bee traffic monitoring of managed colonies, their ultimate success depends on overcoming two challenges: large data engineering and operational energy footprints and ecological and environmental costs of increasing levels of water consumption and electromagnetic radiation. The accuracy vs. energy tradeoff is here to stay. Thus, going forward, it is critical for precision apiculture investigations to evaluate DL models (and other models, too) not only in terms of accuracy, but also in terms of energy efficacy.

## Figures and Tables

**Table 1:** Longitude and latitude of U.S. locations where bee traffic videos were recorded at private and public apiaries and queen races at each location.

City	State	Latitude	Longitude	Queen Race
Logan	Utah	41°44${}^{\prime }$7.76${}^{\prime \prime }$ N	−111°50${}^{\prime }$3.80${}^{\prime \prime }$ W	Carniolan
North Logan	Utah	41°46${}^{\prime }$16.79${}^{\prime \prime }$ N	−111°48${}^{\prime }$34.19${}^{\prime \prime }$ W	Italian
Tucson	Arizona	32°13${}^{\prime }$18.26${}^{\prime \prime }$ N	−110°55${}^{\prime }$35.33${}^{\prime \prime }$ W	Russian
Grass Valley	California	39°13${}^{\prime }$8.62${}^{\prime \prime }$ N	−121°03${}^{\prime }$39.82${}^{\prime \prime }$ W	Italian

**Table 2:** Main training parameters of the models; the complete configuration files are in the yolov3-bees.cfg, yolov4-tiny-bees.cfg, and yolov7-tiny-bees.cfg files in the supplementary materials.

Model	Batch	Width, Height	Learning Rate	Decay	Num Iterations
YOLOv3	16	416, 416	0.00100	0.0005	500,200
YOLOv4-tiny	64	608, 608	0.00261	0.0005	48,000
YOLOv7-tiny	64	416, 416	0.00261	0.0005	144,400

**Table 3:** Training times and energy footprints of models; average loss as reported by Darknet trainer; CPA as reported by Gardner Bender(TM) Power Meter PM3000.

Model	Total Training Time (h)	Average Loss	CPA (kW-h)
YOLOv3	720	0.0881	155.89
YOLOv4-tiny	552	0.1088	119.51
YOLOv7-tiny	192	0.7110	24.990
TOTAL	1464		300.39

**Table 4:** Performance accuracy statistics of trained models; Y3—YOLOv3, Y4T—YOLOv4-tiny, Y7T—YOLOv7-tiny; CH—camera height, TP—true positives, FN—false negatives, FP—false positives, NF—number of frames; NV—number of videos; Precision, Recall, F1, IOU were computed with the formulas in (1).; the values in the TOTAL rows and Precision, Recall, F1, and IOU columns are the means of the CH=1 and CH=2 values in the same columns and the above two rows; the maximal values are bolded.

Model	TP	FN	FP	NF	NV	Precision	Recall	F1	IOU
Y3, CH=1	377	581	0	1800	60	**0.39**	**1.00**	**0.56**	**0.39**
Y3, CH=2	833	2577	0	1800	60	**0.24**	**1.00**	**0.39**	**0.24**
Y3 Total	1210	3158	0	1800	60	**0.28**	**1.00**	**0.43**	**0.28**
Y4T, CH=1	22	748	0	1800	60	0.03	1.00	0.06	0.03
Y4T, CH=2	228	3088	24	1800	60	0.07	0.90	0.13	0.07
Y4T Total	250	3836	24	1800	60	0.06	0.91	0.11	0.06
Y7T, CH=1	271	695	300	1800	60	0.28	0.47	0.35	0.21
Y7T, CH=2	193	3082	498	1800	60	0.06	0.28	0.10	0.05
Y7T Total	464	3777	798	1800	60	0.11	0.37	0.17	0.09

**Table 5:** Cumulative power amounts (CPA) in kW-h of the four data engineering categories; The curation CPA is the same for all models, because it would have taken the same amount of energy to label the images regardless of the number of models; The training CPA is in Table 3; Evaluation I CPA is the amount of power spent on running the models on the 120 evaluation videos and is computed per video processing rates in Table 7; Evaluation II CPA is computed as 9.56/3 kW-h, i.e., the total amount of time it took us to manually evaluate the accuracy of the models on 120 evaluation videos divided by 3.

Model	Curation	Training	Evaluation I	Evaluation II	TOTAL
YOLOv3	11.6	155.89	0.063	3.19	170.74
YOLOv4-tiny	11.6	119.51	0.033	3.19	134.33
YOLOv7-tiny	11.6	24.990	0.018	3.19	39.800

**Table 6:** Energy Efficacy (EFF) of the models computed by the formula in (2) from the values in Table 4 and Table 5 for each performance metric in (1). For example, the Precision EFF of YOLOv3 is $\left(0.28/170.74\right)\times 10^{2}$ ≈ 0.16; the Recall EFF of YOLOv4-tiny is $\left(0.91/134.33\right)\times 10^{2}$≈ 0.68; the F1 EFF of YOLOv7-tiny is $\left(0.17/39.8\right)\times 10^{2}$≈ 0.43; the maximal values are bolded.

Model	Precision EFF	Recall EFF	F1 EFF	IOU EFF
YOLOV3	0.16	0.56	0.25	0.16
YOLOv4-tiny	0.04	0.68	0.08	0.04
YOLOv7-tiny	**0.28 **	**0.93**	**0.43**	**0.23**

**Table 7:** Video processing times per 30-sec MP4 video and power use estimates of the trained models on a GEFORCE GTX-980 GPU computer; monthly energy footprints are based on measurements obtained with Gardner Bender(TM) PM3000 power meters; the monthly footprints of each model are computed as 24×7×4×p, where *p* is the model’s power rate value in the third column.

Model	Time Per Video (s)	Power Rate (kW-h/h)	Monthly Footprint (kW-h)
YOLOv3	9	0.210	141.12
YOLOv4-tiny	5	0.200	134.40
YOLOv7-tiny	3	0.180	120.96

**Table 8:** Estimates of monthly and seasonal operational energy footprints (OEF) of BeePiP in Logan, Utah that consisted of one GEFORCE GTX-980 GPU computer running YOLO3, YOLOv4-tiny, and YOLOv7-tiny (one model per time period) and 10 BeePi loggers recording 30-second MP4 videos every minute 10 h per day and wirelessly transferring each video to the GPU computer; in Northern Utah, the beekeeping season lasts five months (May to September); one BeePi logger’s power rate is 0.003 kW-h/h for an estimated monthly total of 24×7×4×0.0003≈2.02 kW-h. Thus, the estimated monthly OEF of BeePiP with YOLOv3 is 141.12+2.02×10=161.32 and its seasonal OEF is 5×161.32=813.60.

BeePiP	Monthly OEF (kW-h)	Seasonal OEF (kW-h)
BeePiP with YOLOv3	161.32	807.60
BeePiP with YOLOv4-tiny	154.60	773.00
BeePiP with YOLOv7-tiny	141.16	705.80

## Data Availability

Details are in the supplementary materials.

## Supplementary Materials

The following supporting information can be downloaded at: https://www.mdpi.com/article/10.3390/s23156791/s1, Figure S1: **Annotated Figure 1 Left**: An on-hive BeePi logger on top of a 2-super Langstroth hive in Logan, Utah; bottom to top: (1) a landing pad; (2) a light gray super; (3) a blue super; (4) a white super with the BeePi logger hardware; (5) a waterproof plastic box with a Pi camera inside looking down on the landing pad; the box is attached to the front of the third super with two screwed-on metallic brackets; (6) a wooden migratory hive lid on top of the third white super. Figure S2: **Annotated Figure 1 Right**: 2 BeePi loggers on top of 2-super Langstroth hives in Tucson, Arizona; the top boxes on hives contain the logger hardware; water- and dustproof boxes on top of the second supers protect the cameras against rain and dust storms frequent in that area of Arizona. Figure S3: **Annotated Figure 2 Left**: BeePi logger hardware; bottom to top: a Raspberry Pi computer coupled to an 8-megapixel Pi camera; a five terabyte USB disk for archiving data for redundancy in case of GPU computer failures or power supply disruptions; a Pi power charger plugged into a waterproof power cord; videos are wirelessly transferred to a GPU computer over an ad hoc 802.11 local network where they are processed and archived for redundancy in case of logger storage failures. Figure S4: **Annotated Figure 3 Left**: A low-end, low-energy 8-megapixel Raspberry Pi camera v2.1 inside a waterproof camera protection box attached to the front of the super with the on-hive BeePi logger shown in the right picture. Figure S5: **Annotated Figure 3 Right**: An on-hive BeePi logger on top of a one-super hive in Logan, Utah in May 2023; bottom to top: (1) a bottom board with a landing pad; (2) a light gray super; (3) a white super with the logger hardware, and a white waterproof camera protection box with the Pi camera in the left image; (4) a white telescoping hive lid. Figure S6: **Annotated Figure 8 Right**: A GEFORCE GTX-980 GPU computer plugged into a PM3000 power meter. Refs. [50,51,52] mentioned in supplementary materials.

## Abbreviations

CV, AI, DL, ML	computer vision, artificial intelligence, deep learning, machine learning
ConvNet	convolutional neural network or convolutional network
CHMDP	continuous hive monitoring data pipeline
GPU, USB, OS	graphical processing unit, universal serial bus, operating system
OmniBeeM, BeePIV	omnidirectional bee motion, bee particle image velocimetry
CH, CVAT	camera’s height, computer vision annotation tool
GHz, CPA, kW-h	gigahertz, cumulative power amount, kiloWatt-hour
MP4, RGB, fps	MPEG-4, red green blue, frames per second
YOLO	You Look Only Once
TP, TN, FP, FN	true positive, true negative, false positive, false negative
BTP_BFLMTP	Bee True Positive and Bee Flight Motion True Positive
BTP_BCRMTP	Bee True Positive and Bee Crawling Motion True Positive
BTP_BMTN	Bee True Positive and Bee Motion True Negative
BTP_BMFN	Bee Tree Positive and Bee Motion False Negative
BFN_BFLMTP	Bee False Negative and Bee Flight Motion True Positive
BFN_BFLMFN	Bee False Negative and Bee Flight Motion False Negative
BFP	Bee False Positive
EFF, OEF	energy efficacy, operational energy footprint
