# Supplementary material for: Accuracy vs. Energy: An Assessment of Bee Object Inference in Videos from On-Hive Video Loggers with YOLOv3, YOLOv4-Tiny, and YOLOv7-Tiny
Source: Sensors (Basel). 2023 Jul 29;23(15):6791. doi: 10.3390/s23156791 (PMC10422429; doi:10.3390/s23156791)
Supplement: Supplementary file 1 [file sensors-23-06791-s001.zip › sensors-2463661-supplementary.pdf]

Article

Accuracy vs. Energy: An Assessment of Bee Object Inference in Videos from On-Hive Video Loggers with YOLOv3, YOLOv4-Tiny, and YOLOv7-Tiny

Vladimir A. Kulyukin * and Aleksey V. Kulyukin

Department of Computer Science, Utah State University, 4205 Old Main Hill, Logan, Utah, USA
* Correspondence: vladimir.kulyukin@usu.edu

1. Annotated Figures

This section contains the annotated versions of several figures in the article.

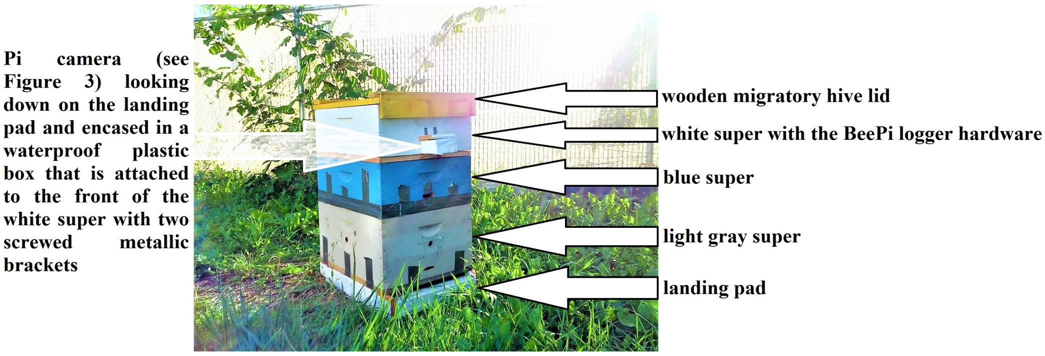

Figure S1. Annotated Figure 1 Left: An on-hive BeePi logger on top of a 2-super Langstroth hive in Logan, Utah; bottom to top: 1) a landing pad; 2) a light gray super; 3) a blue super; 4) a white super with the BeePi logger hardware; 5) a waterproof plastic box with a Pi camera inside looking down on the landing pad; the box is attached to the front of the third super with two screwed-on metallic brackets; 6) a wooden migratory hive lid on top of the third white super.

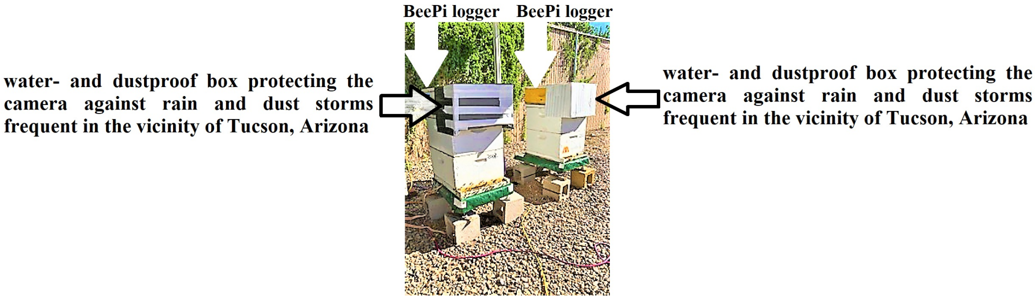

Figure S2. Annotated Figure 1 Right: 2 BeePi loggers on top of 2-super Langstroth hives in Tucson, Arizona; the top boxes on hives contain the logger hardware; water- and dustproof boxes on top of the second supers protect the cameras against rain and dust storms frequent in that area of Arizona.

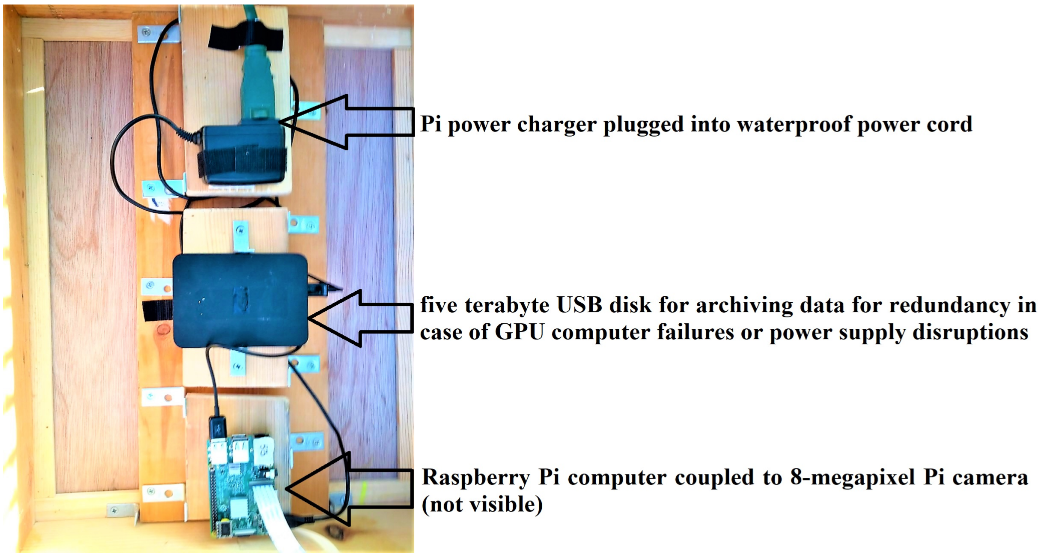

Figure S3. Annotated Figure 2 Left: BeePi logger hardware; bottom to top: a Raspberry Pi computer coupled to an 8-megapixel Pi camera; a five terabyte USB disk for archiving data for redundancy in case of GPU computer failures or power supply disruptions; a Pi power charger plugged into a waterproof power cord; videos are wirelessly transferred to a GPU computer over an ad hoc 802.11 local network where they are processed and archived for redundancy in case of logger storage failures.

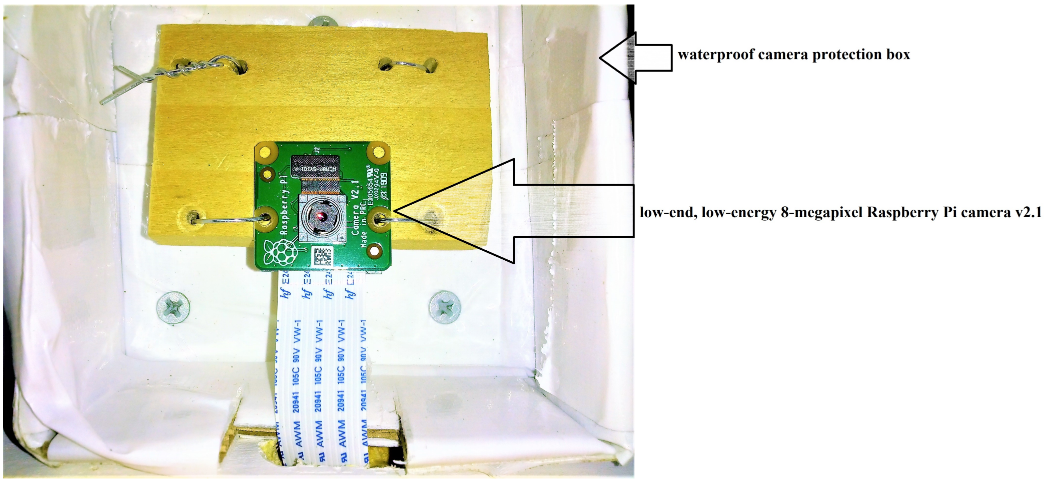

Figure S4. Annotated Figure 3 Left: A low-end, low-energy 8-megapixel Raspberry Pi camera v2.1 inside a waterproof camera protection box attached to the front of the super with the on-hive BeePi logger shown in the right picture.

2. Data Availability

The references below contain the information on the open science fundraisers on www.kickstarter.com [1] executed by the first author and used to partially fund the reported research, our bee image dataset named after Yamuna Lingayya Chatla [2], and the three trained YOLO models [3].

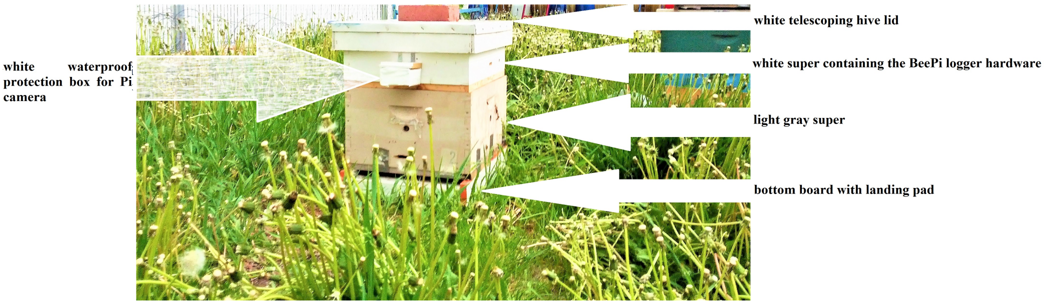

Figure S5. Annotated Figure 3 Right: An on-hive BeePi logger on top of a one-super hive in Logan, Utah in May 2023; bottom to top: 1) a bottom board with a landing pad; 2) a light gray super; 3) a white super with the logger hardware, and a white waterproof camera protection box with the Pi camera in the left image; 4) a white telescoping hive lid.

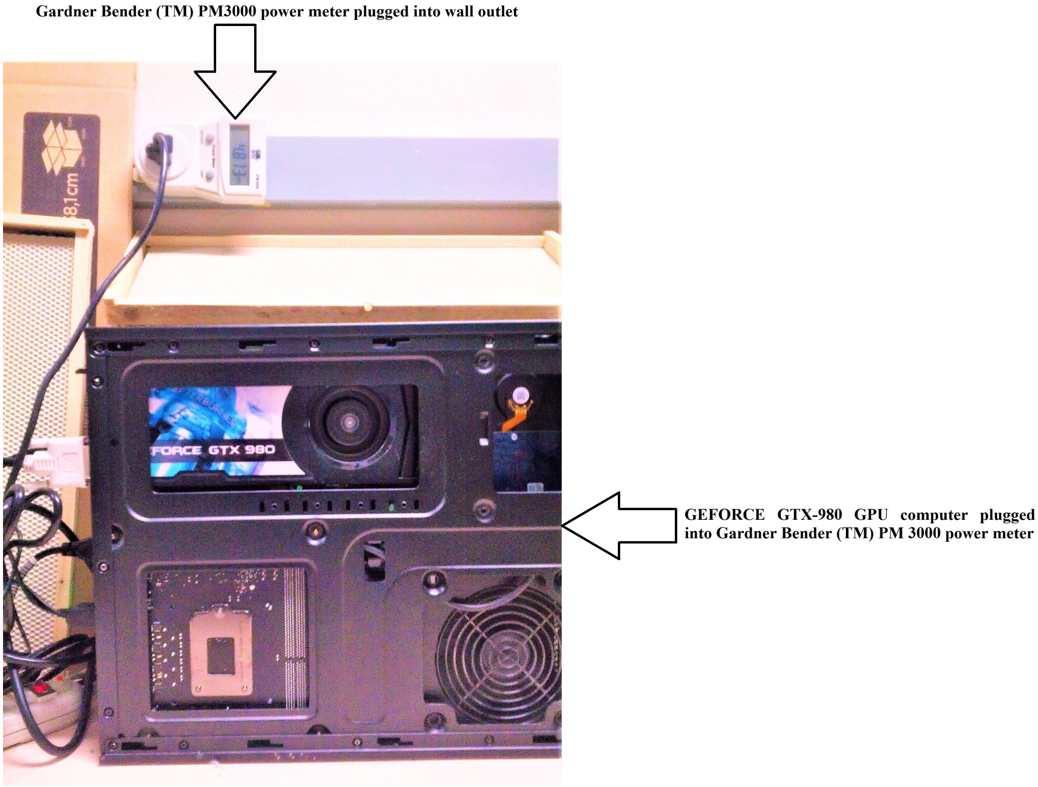

Figure S6. Annotated Figure 8 Right: A GEFORCE GTX-980 GPU computer plugged into a PM3000 power meter.

1. Kulyukin, V. Hive monitoring open science crowdfunders. www.kickstarter.com/profile/beepihoneybeesmeetai/created (accessed on 25 May 2023).
2. Kulyukin, V.; Kulyukin, A. Yamuna Lingayya Chatla image dataset of Apis mellifera objects in ten videos from BeePi on-hive loggers. Yamuna Chatla Bee Image Dataset (accessed on 5 June 2023).
3. Kulyukin, V.; Kulyukin, A. YOLOv3, YOLOv4-tiny, YOLOv7-tiny trained on the image dataset in [2]. Trained YOLO models (accessed on 5 June 2023).
